# Continuity equation-derived valve area using CMR phase-contrast provides flow-independent assessment of valve stenosis

**DOI:** 10.1186/1532-429X-18-S1-P158

**Published:** 2016-01-27

**Authors:** Kevin Gralewski, Walter R Witschey, Sam D Pollock, Kevin K Whitehead

**Affiliations:** 1 Cardiology, The Chidlren's Hospital of Philadelphia, Philadelphia, PA USA; 2 Radiology, Perelman School of Medicine at the University of Pennsylvania, Philadelphia, PA USA

## Background

Assessing aortic valve stenosis (AVS) by cardiac magnetic resonance (CMR) remains challenging, largely due to effects of accelerated flow and resulting pressure recovery, or the phenomena of kinetic energy transmuting to static fluid pressure distally. Effective orifice area (EOA) is a technique used in echocardiography and has been shown to improve AVS assessment. The potential of phase-contrast velocity mapping (PC-MRI) to improve the accuracy of EOA rests on the ability to resolve high peak velocities; previous studies have shown systematic underestimation compared to echocardiography. We used *in vitro* experiments to assess the accuracy of EOA using the continuity equation with PC-MRI derived peak velocities and flows.

## Methods

A custom flow phantom, 20 mm in diameter, was created by 3D printing to mimic AVS by either a 6 mm diameter (D) orifice plate or casted polyurethane bicuspid AV (bAV). Pressure taps were set on the phantom at 20 mm, or 1D, either side of the plate/bAV, 2.5D proximally, and 8D distally. A 40% glycerin-aqueous solution was delivered in various steady-state and pulsatile profiles by an MRI-compatible pump (Shelley Medical Imagining, Ontario, Canada). Pressures were measured by clinical transducers (Edwards Scientific, Washington DC). PC-MRI assessed flow: in-plane acquisitions identified the region of maximal velocity, whereupon optimized through-plane (TR 40 msec, TE 2.3-2.4 ms, 25° flip angle, 1.5 × 1.5 × 4.5 mm) determined peak velocity and flow, the latter taken just proximal to AVS. All PC-MRI data was obtained on Siemens 1.5T Avanto scanner and analyzed by CVI42 (Circle Cardiovascular Imaging, Calgary). EOA in each trial was calculated by the continuity equation (EOA = Peak Flow Rate / Peak Velocity).

## Results

PC-measured average flow rates showed good agreement with programmed Flow rates (SD = 0.07, p < 0.0005). The Bernoilli-derived predicted gradients demonstrated excellent agreement with the measured peak gradient. However, it overestimated the gradient as expected due to pressure recovery. The calculated EOAs were consistent with the physical dimension of the plate/bAV and was flow-rate independent. Conversely, both pressure gradient and pressure recovery were directly related to peak velocity as expected (R^2^= 0.976, p < 0.001; R^2^= 0.946, p < 0.001, respectively, see Fig). Pressure recovery in the plate was also noted to be consistently higher than predictions from previous reports, though this trend did not hold for the bAV trials.

## Conclusions

PC-MR technique can provide accurate peak velocity measurements over a wide range of gradients provided TE is appropriately minimized. As such, PC-MR may provide accurate EOA that is flow-rate independent. Pressure recovery appears multifactorial, though flow and geometry of stenosis seem to be the most significant. PC-MRI derived EOA is a promising technique that may provide more reliable quantification of valve stenosis than echocardiography due to more accurate flow measurement and less variability.

**Figure 1:**
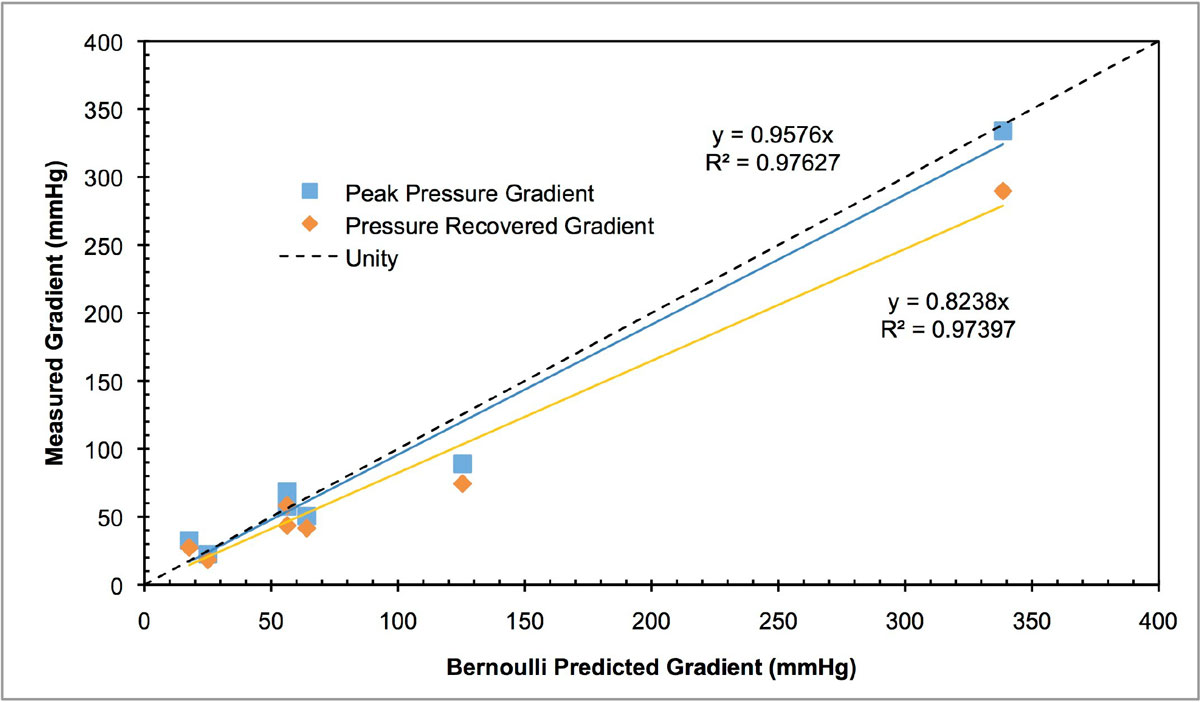
**The linear character of the peak pressure gradient across the orifice/valve and the recovered pressure gradient, both as a function of the respective gradients predicted by the continuity equation from Bernoulli principle**.

